# Stroke in Patients with Atrial Fibrillation: Epidemiology, Screening, and Prognosis

**DOI:** 10.3390/jcm13010030

**Published:** 2023-12-20

**Authors:** Olli Pekka Suomalainen, Nicolas Martinez-Majander, Jenna Broman, Laura Mannismäki, Aapo Aro, Sami Curtze, Sami Pakarinen, Mika Lehto, Jukka Putaala

**Affiliations:** 1 Departments of Neurology, Helsinki University Hospital, University of Helsinki, Haartmaninkatu 4, P.O. Box 340, 00290 Helsinki, Finland; nicolas.martinez-majander@hus.fi (N.M.-M.); jenna.broman@hus.fi (J.B.); laura.mannismaki@hus.fi (L.M.); sami.curtze@hus.fi (S.C.); jukka.putaala@hus.fi (J.P.); 2 Departments of Cardiology, Helsinki University Hospital, University of Helsinki, 00290 Helsinki, Finland; aapo.aro@hus.fi (A.A.); sami.pakarinen@hus.fi (S.P.); mika.lehto@hus.fi (M.L.)

**Keywords:** atrial fibrillation, hemorrhagic stroke, ischemic stroke, oral anticoagulation, prevention, screening

## Abstract

Atrial fibrillation (AF) is the most common sustained arrythmia and one of the strongest risk factors and causal mechanisms of ischemic stroke (IS). Acute IS due to AF tends to be more severe than with other etiology of IS and patients with treated AF have reported to experience worse outcomes after endovascular treatment compared with patients without AF. As cardioembolism accounts for more than a fifth of ISs and the risk of future stroke can be mitigated with effective anticoagulation, which has been shown to be effective and safe in patients with paroxysmal or sustained AF, the screening of patients with cryptogenic IS (CIS) for AF is paramount. Embolic stroke of undetermined source (ESUS) is a subtype of CIS with a high likelihood of cardioembolism. The European Stroke Organization and European Society of Cardiology guidelines recommend at least 72 h of screening when AF is suspected. The longer the screening and the earlier the time point after acute IS, the more likely the AF paroxysm is found. Several methods are available for short-term screening of AF, including in-hospital monitoring and wearable electrocardiogram recorders for home monitoring. Implantable loop monitors provide an effective long-term method to screen patients with high risk of AF after IS and artificial intelligence and convolutional neural networks may enhance the efficacy of AF screening in the future. Direct oral anticoagulants (DOACs) are preferred over vitamin K antagonists in both primary and secondary prevention of IS in AF patients. Recent data from the randomized controlled trials (RCT) also suggest that early initiation of DOAC treatment after acute IS is safe compared to later initiation. Anticoagulation treatment may still predispose for intracranial bleeding, particularly among patients with prior cerebrovascular events. Left atrial appendix closure offers an optional treatment choice for patients with prior intracranial hemorrhage and may offer an alternative to oral anticoagulation even for patients with IS, but these indications await validation in ongoing RCTs. There are still controversies related to the association of found AF paroxysms in CIS patients with prolonged screening, pertaining to the optimal duration of screening and screening strategies with prolonged monitoring techniques in patients with ESUS. In this review, we summarize the current knowledge of epidemiology, screening, and prognosis in AF patients with stroke.

## 1. Introduction

Stroke remains among the leading causes of mortality and long-term disability globally [1,2]. Atrial fibrillation (AF), in turn, is the most common sustained arrhythmia and one of the strongest risk factors and causal mechanisms for ischemic stroke (IS) [3,4,5,6,7]. Cardioembolic causes account for 20–25% of all IS patients, with known AF being by far the most frequently documented source for cardioembolism [4,8,9,10,11]. The incidence of AF increases strongly with age, leading to a prevalence of almost 20% in a population aged more than 85 years [9,12]. As AF can account for at least a fifth of cases with IS and the strokes caused by AF tend to be more severe than other types of IS, AF is a major modifiable risk factor for IS, including disabling stroke [4,8,9]. Anticoagulation in patients with IS and AF has shown to be effective and safe [4,13]. The flipside of the coin is that anticoagulation can also lead to both hemorrhagic stroke and hemorrhagic transformation (HT) of IS in patients with AF [4].

**Figure 1:**
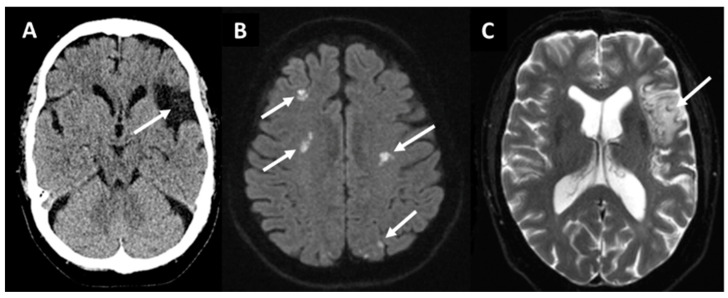
Non-contrast computed tomography (**A**) showing a chronic embolic stroke of undetermined source (arrow) in a 66-year-old woman in the left middle cerebral artery territory. Magnetic resonance diffusion-weighted imaging (**B**) revealing ischemic lesions (bright spots indicated by arrows) in multiple territories in a 78-year-old man with chronic atrial fibrillation. T2*-weighted imaging (**C**) showing a subacute lesion (arrow) in the left middle cerebral artery territory in a patient with paroxysmal atrial fibrillation.

In one third of IS patients, the etiology of stroke remains cryptogenic despite standard diagnostic evaluations and, in such circumstances, screening for occult paroxysmal AF is recommended [4,10,14]. Brain imaging findings of IS patients raising a suspicion of cardioembolism include an acute or chronic ischemic cortical, but not lacunar lesions, multi-territorial (cortical) lesions with a high prevalence in posterior circulation and with possible HT [11,15,16]. Magnetic resonance imaging (MRI) can be used to reveal the embolic pattern of IS which is not shown in non-contrast computed tomography (NCCT) scans (Figure 1B) [16].

**Figure 2:**
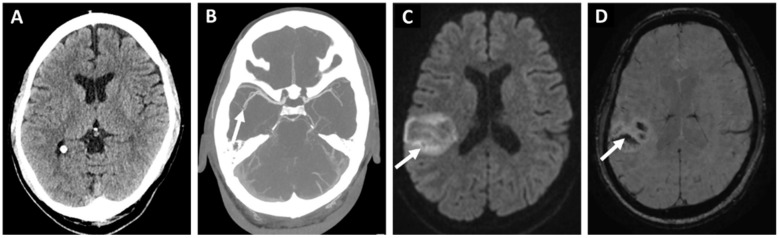
76-year-old woman with hypertension and dyslipidemia presented with a sudden onset left hemiparesis and dysarthria. Initial non-contrast computed tomography (**A**) showed no acute hypodensity. The patient received immediate intravenous thrombolysis after which computed tomography angiography (**B**) revealed a right-sided middle-cerebral artery occlusion in the M2 segment too distal to be treated with endovascular treatment. Despite thrombolysis, the next day magnetic resonance diffusion-weighted imaging (**C**) showed a cortical infarction (arrow) with a moderate hemorrhagic transformation in the susceptibility weighted imaging (**D**). Etiologic classification for the stroke was embolic stroke of undetermined source (arrow) after initial diagnostic work-up. And implantable loop monitor inserted 11 weeks later revealed a paroxysmal atrial fibrillation as the probable source of the embolism.

The embolic stroke of undetermined source (ESUS) concept defines a subclass of cryptogenic IS (CIS) with a higher likelihood of detecting occult AF through prolonged monitoring (Figure 1 and Figure 2) [11,17]. The European Society of Cardiology (ESC) and European Stroke Organization (ESO) guidelines recommend that patients with CIS or transient ischemic attack (TIA) should undergo at least 72 h of ECG monitoring, with further prolonged screening after initially negative screening being recommended [13,18]. It is well documented that longer screening leads to an increased rate of positive AF paroxysms [4,13,18,19,20].

Oral anticoagulation (OAC), either with vitamin K antagonists (VKAs) in valvular AF or direct oral anticoagulants (DOACs) in non-valvular AF effectively mitigates the risk of recurrent IS in patients with AF. Recent data suggest that early initiation of DOAC after TIA or IS due to AF is safe compared to later initiation. However, OAC may predispose some patients for intracranial bleeding, including intracerebral hemorrhage (ICH), subarachnoid hemorrhage, and traumatic intracranial hemorrhage [3,5]. The association of silent cerebral microbleeds (CMBs) with occult AF and the role of anticoagulation in AF patients with CMBs is still unclear [8]. Left atrial appendix closure (LAAC) offers an option for anticoagulation treatment as the primary or secondary prevention of thromboembolism in AF patients with anticoagulation-related spontaneous intracranial bleeding or other contraindication for anticoagulation or in breakthrough IS, i.e., despite proper anticoagulation treatment. However, it is still unclear whether these patients should be left untreated or treated with aspirin, VKA/DOAC, or with LAAC [8].

In this review, we summarize the current knowledge of epidemiology, screening, and prognosis in patients with stroke and AF.

## 2. Epidemiology and Clinical Features

### 2.1. Burden of AF and Factors Predisposing to AF

Incidence and prevalence of AF are distinctly age-related, and as the population ages, the global burden of AF is increasing remarkably [21,22,23]. In a Finnish nation-wide study, the increase in AF incidence was strongly age-dependent, with age-standardized incidence remaining stable during 2007–2018 [12]. In the European Union, 8.8 million individuals over 55 years were estimated to have AF in 2010, which is predicted to double to 17.9 million by 2060 [24]. Globally, during the next 30 years, the estimated burden of AF has been predicted to increase by 66%, reaching 62.5 million cases [25]. Both awareness and enhanced detection of AF have improved over the past decade, as has survival of individuals with AF [22].

Genetic predisposition has been recognized as a major risk factor for AF (Table 1) [21]. Both the prevalence and incidence of AF are higher in men, compared to women, and both are higher in high-income compared to low-to-middle income countries [21]. Also, European ancestry has been associated with a higher risk of AF in African Americans [26]. The prevalence of AF increases remarkably with advancing age, the most important risk factor for AF [22], as well as with cardiovascular comorbidities, reaching up to 17% among adults aged 80 or older [12,27,28]. Vascular and myocardial aging involve changes at the cellular, molecular, structural, and functional levels, predisposing patients to AF (Table 1) [22]. Moreover, AF is associated with valvular heart disease, chronic heart failure, thyroid disorders, and recent heavy alcohol use [29]. The modifiable risk factors predisposing to development and perpetuation AF include also smoking, body mass index, diabetes, obstructive sleep apnea, and myocardial infarction [23]. The largest population-wide attributable risk factor for AF is hypertension [22], which can lead to left atrium (LA) and left ventricular (LV) structural remodeling and engages in prothrombotic changes [30].

### 2.2. Mechanism and Typical Clinical Picture of IS Due to AF

In randomized clinical trials, the average annual risk of all-type stroke was about 1.5% in AF patients [31]. The causal association of AF and IS has been supported by a series of cohort-based studies [32]; however, the precise underlying mechanisms between the pathophysiological elements, AF, and stroke are not fully understood [4]. In part, despite the clear association between AF and IS, AF might also represent a marker of cardiovascular burden in the aging population [33]. According to existing data, the abnormal changes in flow, vessel wall, and blood constituents involved in AF fulfil Virchow’s triad for thrombogenesis and thus AF could lead to a prothrombotic or hypercoagulable state [4]. However, the mechanisms underlying thrombogenesis in AF leading to cardioembolic stroke are complex and only partly understood [34]. In patients with cardiac implantable devices, AF was associated with an increased risk of IS and embolism with rather long AF-episodes (>24 h), whereas only a few patients had subclinical AF in a temporal relation with a stroke or an embolism [35,36]. Therefore, additional factors contributing to AF-related strokes have been researched. This has led to the concept of atrial cardiomyopathy seen in animal models and in patients with AF, where electrical and structural remodeling in the atrium is caused by a complex interplay of AF, atrial fibrosis, and other factors [35]. However, whether atrial fibrosis is the causative factor of AF or rather a marker of underlying disease, and whether atrial fibrosis contributes to IS remains unclear, since the relationship between atrial fibrosis, AF, and stroke is not yet fully understood [35].

AF-related IS is almost twice as likely to be fatal, is usually more severe, and recurs more often than the IS of other etiologies [36,37,38]. Clinical features implying a cardioembolic IS etiology include sudden (<5 min) onset to maximal deficit, a Valsalva maneuver at the time of stroke onset, decreased consciousness level on the stroke onset, Wernicke’s aphasia or global aphasia without hemiparesis, rapid regression of the symptoms, and the co-occurrence of cerebral and systemic emboli [29]. Furthermore, neuroimaging findings supporting IS of cardioembolic origin are simultaneous or sequential strokes detected in different arterial territories, HT, which occurs in between 3.2 and 43.3% of all strokes and of which the majority are cardioembolic strokes, and the early recanalization of an occludedintracranial vessel [29,39]. The annual risk of recurrent IS in AF patients without OAC was considerably high in early studies, even in patients with few comorbidities. For example, for a CHA_2_DS_2_-VASc-risk score of 2, the annual risk of recurrence was 2.49% (95% confidence interval, CI 1.16–3.83%), and for a score of 1, the risk was 1.61% (95% CI 0–3.23%) [40,41,42]. In previous historical studies, the risk of recurrent IS and mortality is worst in patients with cardioembolic IS compared to other etiologies of IS [43].

### 2.3. Risk Stratification Schemes

The mainstay of the prevention of AF-related stroke, OAC, comprising VKA and DOACs, significantly reduces the risk of stroke, as well as mortality [44,45,46,47,48]. The overall risk for stroke in AF is five-fold; however, the risk is not homogenous, but depends on the presence of specific stroke risk factors [13]. Moreover, persistent AF increases the risk of stroke compared to paroxysmal AF [49]. The most common risk scale for AF-related stroke is CHA_2_DS_2_-VASc [13,40,50], which summarizes the common stroke risk factors: congestive heart failure, hypertension, age ≥ 75 years, diabetes mellitus, stroke, vascular disease, age 65–74 years, and sex category (female). Patients identified as low-risk (those having a CHA_2_DS_2_-VASc score of 0 for men and 1 for women), with an annual rate of stroke of less than 1%, do not need any antithrombotic (AT) therapy, whereas any score above that triggers the requirement to consider OAC [13]. Patients with IS all belong to at least a moderate-risk group since they have a considerably increased risk of recurrent IS and other thromboembolic events. Patients with OAC-associated ICH (OAC-ICH) have previously shown to have larger hematomas than patients with non-OAC associated ICH [51].

However, when initiating AT, an evaluation of bleeding risk utilizing a validated bleeding risk score such as HAS-BLED (uncontrolled hypertension, abnormal renal and/or hepatic function, stroke, bleeding history or predisposition, labile INR, elderly, drugs or excessive alcohol drinking) score is also recommended [52]. Acknowledging the clear clinical benefit of OAC, the bleeding risk scores are recommended to be used as tools for more efficient management of the modifiable risk factors for bleeding and identifying patients in need of more frequent reviews, rather than for withholding OAC [13]. 

### 2.4. AF and Hemorrhagic Stroke

AF is a known risk factor for HT in acute IS patients [53,54,55]. Cardioembolism is a frequent cause of spontaneous, hemorrhagic infarcts and, furthermore, HT, which is divided into hemorrhagic infarction and parenchymal hemorrhage with or without mass effect, also occurs in up to 11% of cardioembolic strokes [56,57]. HT within a week after IS was observed in up to 9% of patients, and 25.8% of those were associated with the presence of AF [54].

Furthermore, the most feared complication of long-term systemic OAC in AF patients is intracranial hemorrhages, which account for nearly a quarter of all ICHs altogether and are known to be associated with a high risk of in-hospital mortality and poor outcomes [58,59]. Approximately 70% of OAC-ICH are observed in patients with underlying cerebral small vessel disease. Neuroimaging findings (severe white matter hyperintensities, CMBs, and cortical superficial siderosis) can be used to identify AF patients’ underlying hemorrhage-prone pathologies, including hypertensive small vessel disease and amyloid angiopathy [58].

A short time in the therapeutic range of VKA-patients, antiplatelet medication in addition to anticoagulation treatment, and chronic renal disease are some known risk factors that have been shown to predispose for OAC-ICH [60]. Baseline ICH volume, hematoma expansion, 90-day mortality, and functional outcome have been shown to be similar between DOAC-ICH and VKA-ICH [61].

### 2.5. Cryptogenic IS and ESUS

Symptomatic cerebral infarcts with no probable cause identified after sufficient diagnostic evaluation are defined as cryptogenic IS (CIS) [62]. Such cases account for up to one third of patients [10,17]. A subgroup of patients with CIS, in which, despite an extensive diagnostic workup, no potential cause is recognized, is described as ESUS (Table 1) [11]. The diagnosis of ESUS requires adequate investigations to exclude a major-risk cardioembolic source, proximal occlusive atherosclerosis, as well as lacunar infarcts caused by cerebral small vessel disease [11].

ESUS implies a proximal, often cardiac, embolic mechanism and covert paroxysmal AF is a potential cause of CIS [11]. Due to its dynamic nature, identifying occult AF may be challenging in clinical practice, although it may be present in a significant proportion of patients presenting with cryptogenic IS. When using extended cardiac monitoring (up to 3 years), AF (at least 30 s paroxysm) can be detected in 30–40% of these patients [11,63,64,65].

Although occult AF and atrial cardiomyopathy may be a potential source of embolism in ESUS patients, other factors including low-degree atherosclerotic stenosis, paradoxical embolism through patent foramen ovale, left ventricular disease or heart failure, and occult malignancies may serve as potential embolic sources, which frequently overlap [66]. In IS or TIA patients, in addition to increasing age [18], suspected cardioembolic sources based on clinical and radiological evaluation predict AF [58]. 

## 3. Screening

### 3.1. AF Screening in the General Population to Prevent IS

In many patients, IS may be the first symptom of undetected AF, with a high mortality if IS and AF occur concurrently [67]. Thus, there have been efforts to actively identify those with asymptomatic or non-documented AF and stroke risk factors for whom a new diagnosis would lead to appropriate treatment, e.g., the initiation of OAC and enhanced management of both AF and stroke risk factors. Early diagnosis of AF may also enable early rhythm control therapies, which have been associated with a lower risk of adverse cardiovascular outcomes in symptomatic and asymptomatic patients [68,69]. Screening strategies for AF vary depending on the population studied (e.g., patients with CIS vs. individuals from the general population). 

Screening may be performed intermittently with pulse palpation, photoplethysmography (PPG) recorders (e.g., smart watches or mobile phones) or single-lead ECG, which should always be used as confirmatory method for the former. Continuous monitoring devices include traditional Holter, loop ECG recorders, ECG patches, chest strap ECG recorders capable of monitoring for various durations, and implantable loop monitors (ILMs). The Apple Heart Study using the Apple Watch and the Fitbit Heart Study using the Fitbit smart watch, detected patients with likely AF paroxysms for further screening with ECG patch monitoring in the general population [70,71]. The ongoing Rhythm Evaluation for AntiCoagulaTion with Continuous Monitoring of Atrial Fibrillation (REACT-AF trial, NCT05836987) uses the Apple Watch and compares standard DOAC versus time-limited (1 month) DOAC treatment in patients with detected AF paroxysms. In addition, cardiac pacemakers with an atrial lead are also capable of monitoring atrial rhythm. Positive AF detection rates depend strongly on the screening setting, target population, and duration of monitoring, and can vary from <1% in non-selected cohorts to >7% in patients at a higher risk [72]. Increasing the duration and frequency of screening measurements will obviously also increase AF detection rates [18]. Previous meta-analyses have shown that, in patients with a high risk of IS, the incidence of IS was significantly less frequent omitted in patients with prolonged cardiac screening; therefore, representing an effective stroke prevention strategy in selected patients [73].

Three randomized controlled trials have been published on the implications AF detected by screening or implantable cardiac device. In the STROKESTOP study, individuals were randomized to intermittent twice-daily ECG screening for two weeks or regular care [74]. In the STOKESTOP 2 study, N-terminal B-type natriuretic peptide-stratified systematic screening for AF identified 4.4% of the high-risk participants with new AF [75]. During the follow-up of 6.9 years, a small benefit on the combined endpoint of stroke, mortality and major bleeding was detected. In the LOOP study (ILR monitoring vs. usual care), AF was diagnosed in 31.8% of the participants in the screened group, but there was no significant reduction in the primary outcome of stroke or systemic embolism [76]. Most recently, the non-vitamin K antagonist oral anticoagulants in patients with atrial high rate episodes (NOAH-AFNET 6–trial) studied whether the occurrence of atrial high-rate episodes (AHRE ≥ 6 min), resembling AF detected by implanted cardiac devices, justifies the initiation of anticoagulation in patients with at least one additional stroke factor [77]. The trial was terminated early due to futility, as anticoagulation did not reduce the combined end point of stroke and cardiovascular death, but led to a higher incidence of a composite of death or major bleeding. Taken together, these studies indicate that in high-risk populations long-term cardiac screening may result in a lower risk of first and recurrent IS [73], but due to existing uncertainties, systematic screening is not yet strongly recommended and should be targeted at individuals aged ≥75 years or those at high risk of stroke [13,73]. Opportunistic screening is, however, strongly recommended in all individuals aged ≥65 years [13].

### 3.2. AF Screening in Cryptogenic IS

Many ESUS patients may have had undiagnosed AF, which is why it is recommended that CIS patients should undergo at least 72 h of ECG monitoring [13,18]. Importantly, anticoagulation of all ESUS patients without diagnosis of AF is not recommended based on the negative results of the NAVIGATE ESUS [78] and RE-SPECT ESUS [79] trials. This observation has prompted further efforts to identify those ESUS patients who may have had a cardioembolic stroke presumably due to AF.

CIS patients are often screened with baseline ECG, telemetry, or with other continuous cardiac monitoring while hospitalized, and with non-invasive cardiac monitoring such as episodic ECG and ambulatory ECG [65,76,80]. As a more invasive method, ICMs can be used to detect AF and to screen ESUS-type stroke patients for occult AF [81]. By combining different screening methods at different stages, including wearable ECGs and ICMs, a cumulative rate of new AF, as high as 23.7%, can be achieved in IS patients with no previous diagnosis of AF [82]. This rate could be even higher if screening is focused strictly on CIS or ESUS patients. 

The longer scanning time of ICMs leads to increased rates of AF paroxysms, but the causal relationship of the detected AF paroxysms with IS is still unclear, as is the optimal screening strategy and scanning duration [19,80,83]. However, there is accumulating evidence that initiating ECG screening early after IS is more efficacious compared to later initiated screening, and that certain screening methods, including chest belt ECG, may be more effective in the early stage of screening [84]. Moreover, there is a relatively strong consensus view on the benefits and cost-efficacy of the staged AF screening strategy after cryptogenic IS [85]. In our view, the ESUS patient population is the optimal target for AF screening. Furthermore, efforts should be made to validate different screening techniques and strategies as we have, for example, validated devices utilizing either plaster electrodes or chest strap electrodes in patients with recent ESUS [86,87].

### 3.3. Detection of Atrial Tachyarrhythmia’s with Pacing Devices

Monitoring various forms of atrial tachyarrhythmia (ATA), such as AF, atrial flutter, and atrial tachycardia, has become a crucial application for pacing device diagnostics. Precise recognition of atrial tachyarrhythmias (ATA) in the form of atrial high-rate episodes (AHREs) using cardiac implantable electronic devices (CIED) enables the strategic guidance of optimal anticoagulation and anti-arrhythmic therapy. The accuracy of detecting atrial high-rate episodes (AHRE) relies on the proper sensing and discrimination of atrial potentials [88,89,90].

Intermittent undersensing of continuous AF and inadequate sensing of very brief episodes of AF or other ATAs are frequently observed and may lead to inappropriate AHRE detection [90,91,92]. An erroneous AHRE detection may be triggered by oversensing of the far field R-wave [93] or sensing of retrograde atrial depolarizations [92,94,95]. In the Atrial Fibrillation Reduction Atrial Pacing (ASSERT) trial, 17.3% of the AHREs were inaccurately detected, which was primarily attributed to repetitive non-reentrant ventriculo-atrial synchronous rhythm [94].

Device-detected AHREs without apparent symptoms have been linked to an elevated risk of IS, mortality, and a higher burden of AF [96,97,98]. In individuals receiving pacemakers with no prior history of AF, 35–72% of all IS or systemic embolisms were preceded by AHRE detected by CIEDs [96,97,98]. Significantly, a proximate temporal relationship between device-detected AHREs and the onset of IS has not been demonstrated in the majority of patients [99,100,101]. Currently, there is a lack of evidence either supporting or opposing the prescription of OAC in patients at an elevated risk of IS who exhibit AHREs.

Numerous studies have convincingly demonstrated the association between device-detected ATAs and the occurrence of stroke or systemic thromboembolism in patients with cardiac implantable devices [96,97,98]. The recent Apixaban for the Reduction of Thrombo-Embolism in Patients with Device-Detected Sub-Clinical Atrial Fibrillation (ARTESiA) study, which assessed the effectiveness and risk–benefit balance of OAC compared to no OAC (with aspirin alone as the comparator) in patients exhibiting device-detected ATAs, showed that the incidence of IS was low in patients with AHRE episodes and OAC with apixaban did not significantly reduce the incidence of a composite of cardiovascular death, stroke, or systemic embolism as compared with placebo in these patients [102]. A recent meta-analysis combining the results from the ARTESiA and NOAH-AFNET 6 trials also suggests that the risk of IS with AHRE-episodes is significantly lower than with clinical episodes of AF [103].

## 4. Primary and Secondary Prevention of IS in Patients with AF

Earlier studies have shown that, compared to non-AF IS, IS associated with AF is likely to be more severe and cause greater disability, fatality, and costs [7,104,105]. These patients are usually older and AF-associated IS most commonly causes larger tissue damage and tends to present in the middle cerebral artery territory. However, research thus far has mostly assessed short-term recovery and mortality after AF-associated stroke, and contemporary data on the long-term outcomes is scarce. Older follow-up studies from almost 30 years ago have reported up to a two-fold mortality in stroke patients with AF compared to patients with no AF. For example, in the Copenhagen Stroke Study, the odds ratio for 30-day mortality was 1.7 (95% CI 1.2–2.5), and in the Framingham Study 1.8 (95% CI 1.1–3.3) [36,37]. Furthermore, some studies reported an increased risk of death being highest within the first weeks after AF-associated IS [106]. The risk of annual recurrence has been suggested to be up 10%, even in anticoagulated AF patients, and the mortality up to 50% within 2 years after the index stroke [107,108].

The risk of IS in AF patients can be reduced with appropriate selection of OAC, either with warfarin or DOACs (i.e., rivaroxaban, apixaban, edoxaban, or dabigatran). Based on indirect estimates, anticoagulation reduces the risk by approximately two thirds (60–70%) compared to placebo, regardless of the baseline risk [109]. Notably, OAC use also reduces the severity of IS, results in lower rates of large vessel occlusion and in a better 3-month functional outcome after IS, compared to patients with AF and no OAC at the time of the stroke [110]. Current guidelines recommend DOACs over vitamin K antagonists as the first-line stroke prevention in patients with non-valvular AF [13,111]. In a meta-analysis of four pivotal randomized controlled landmark trials (Randomized Evaluation of Long-Term Anticoagulant Therapy (RE-LY), Rivaroxaban versus Warfarin in Nonvalvular Atrial Fibrillation (ROCKET AF), Apixaban versus Warfarin in Patients with Atrial Fibrillation (ARISTOTLE), and Edoxaban versus Warfarin in Patients with Atrial Fibrillation (ENGAGE AF)) investigating DOACs, DOACs were associated with a significant 19% reduction in stroke/systemic embolism, a favorable risk–benefit profile, and a similar IS risk reduction compared with warfarin, although with increased gastrointestinal bleeding [112]. The relative efficacy and safety of new oral anticoagulants was consistent across a wide range of patients. Moreover, DOACs possessed a 52% reduction in intracranial hemorrhage compared with warfarin, and a 10% reduction in all-cause mortality [44]. However, DOACs are not recommended in patients with mechanical heart valves—for instance, the randomized phase II study to evaluate the safety and pharmacokinetics of oral dabigatran etexilate in patients after heart valve replacement (RE-ALIGN) trial compared dabigatran versus warfarin in valvular AF but was prematurely stopped due to a high rate of adverse events, both thromboembolic and bleeding, in the dabigatran arm [113]. Finally, a newer class of OACs, factor XIa inhibitors, is under investigation. These include asundexian, which in the safety of the oral factor Xia inhibitor asundexian compared with apixaban in patients with atrial fibrillation (PACIFIC-AF) phase 2 trial, was shown to be well tolerated and had a lower risk of bleeding compared with apixaban [114]. Phase 3 trials of factor XIa are currently running.

There are no randomized trials assessing the safety and efficacy of different anticoagulation regiments specifically in the setting of secondary prevention in AF patients who have experienced a prior IS or TIA. Nevertheless, a pooled analysis of randomized trials in the ESO guidelines on AT treatment for secondary prevention of stroke and other thromboembolic events in patients with IS or TIA and non-valvular AF concluded that DOACs are superior in safety and at least as efficacious as warfarin in secondary prevention [115]. Many patients with AF also have concomitant coronary artery disease and AT therapy is often administered in conjunction with OAC therapy or as substitution. The Global Anticoagulant Registry in the Field-Atrial Fibrillation (GARFIELD-AF) trial, studying AF patients with or without acute coronary disease, showed that previous acute coronary syndromes were associated with worse 2-year outcomes, major bleeding, and a greater likelihood of undertreatment with OAC, while two thirds of patients received AP therapy [116].

### 4.1. Timing of OAC Initiation after IS and Its Impact on Short-Term Outcomes

Data on the optimal timing for OAC initiation after IS is still relatively limited. Traditionally, initiation of OAC has usually been delayed, reducing the risk of HT. However, the risk of HT must be balanced with the increasing risk of recurrent thromboembolism, especially within the first days to weeks after the index stroke. In 2013, the European Heart Rhythm Association suggested a 1–6–12-day rule, i.e., the number of days from the occurrence of IS to OAC initiation based on the stroke severity measured with the NIH stroke scale [117]. Similar recommendations have been published by ESO and the American Heart/Stroke Association (AHA/ASA). Until recently there has been no high-quality RCT on this topic and each recommendation was based on expert opinions. However, two very recent trials, the Early Versus Delayed Non-Vitamin K Antagonist Oral Anticoagulant Therapy After Acute Ischemic Stroke in Atrial Fibrillation (TIMING) trial and the Early Versus Late Initiation of Direct Oral Anticoagulants in Post-Ischaemic Stroke Patients with Atrial Fibrillation (ELAN) trial, reported that early initiation of DOAC was non-inferior to a delayed start with no safety concerns [118,119]. In the ELAN trial, the 30-day recurrent IS rate was 1.4% in the early treatment vs. 2.5% in the late treatment group (OR 0.57; 95% CI 0.29–1.07). In the ELAN trial, the early initiation of OAC after IS ranged from 48 h to 6–7 days, depending on stroke severity (minor, moderate, and large IS). Neither the ELAN nor the TIMING trial included patients with such parenchymal hemorrhage or other cause that the treating clinician did not permit randomization. These results implied that early initiation of DOAC should be considered in patients eligible for DOAC treatment. Notably, bridging with low-molecular weight heparins cannot be recommended before starting OAC [120].

### 4.2. LAA

A recent prospective multicenter observational study showed that patients with AF suffering from IS despite being on DOAC (“breakthrough IS”) have a high risk of recurrent stroke and bleeding during a mean follow-up time of almost 16 years [121]. The rates did not differ in patients who changed or did not change from one DOAC to another. Patients with IS while using OACs are shown to have an increased risk of all-cause death and further stroke recurrences (both ischemic and hemorrhagic) [110]. Breakthrough strokes are not uncommon in clinical practice, and they are associated with poor prognosis, while the mechanism of underlying OAC failure is still not completely understood, making the prevention of recurrent events a challenge without proper guidelines [122]. In these patients, there is no evidence to support switching from one DOAC to another or between a good-quality vitamin K antagonist and DOAC (or vice versa), regarding the risk of recurrent IS [123,124]. In such cases, LAAC, also with lower bleeding risks, might be considered as an alternative treatment strategy, but the overall benefit still remains unclear. An ongoing Left Atrial Appendage Occlusion Versus Novel Oral Anticoagulation for Stroke Prevention in Atrial Fibrillation Multicenter Randomized Clinical Trial (Occlusion-AF) compares DOACs with LAAC in patients with a recent (less than 6 months) ischemic stroke. In addition, other studies on the breakthrough IS population are on the way (Table 2) [122].

## 5. Acute Recanalization Treatment and Its Outcomes after AF-Associated IS

Patients with AF treated with endovascular treatment for acute IS have been reported to experience worse outcomes compared with patients without AF. In a recent systematic review and meta-analysis including patients with AF and acute IS, patients with AF and large vessel occlusion experienced worse 90-day outcomes and higher mortality compared to those without AF, even in the setting of similar rates of successful reperfusion [125]. This was likely associated with advanced age and a greater rate of comorbidities among patients with AF.

## 6. AF and Hemorrhagic Stroke

AF patients suffering from intracerebral hemorrhage during VKA treatment should be treated as acutely as possible with a 4-factor prothrombin complex concentrate to reverse the effect of VKA and prevent hematoma growth and clinical deterioration. This should be given together with intravenous vitamin K to re-establish vitamin K-dependent coagulation factor production [126]. In ICH patients with DOACs, either idarucizumab (direct thrombin inhibitors) or andexanet alfa (factor Xa inhibitors) can be considered [126]. However, evidence on the efficacy of andexanet alfa for functional recovery in ICH patients is still limited and the reversal agent is costly. When specific agents are not available, 4-factor prothrombin complex can be considered, albeit, again, evidence is limited in DOACs. The recently stopped Andexanet Alfa in ICrH Patients Receiving an Oral FXa Inhibitor (ANNEXa-I) trial achieved its pre-specified criteria on hemostatic efficacy but results regarding efficacy on clinical outcomes are still awaited (the abstract was presented at the 2023 World Stroke Congress; (WSC 2023 Late breaking abstracts https://journals.sagepub.com/doi/10.1177/17474930231201072, accessed on 30 November 2023) the overall outcome of patients with either VKA or DOACs is still poorer than in patients without OAC, and in-hospital mortality can still be up to 42% in VKA patients despite effective reversal of warfarin [127].

Randomized evidence is still lacking on the benefits and optimal timing of OAC resumption after hemorrhagic stroke, and no clear recommendations for this matter exist to date. Most often this must be evaluated multi-disciplinarily as an individual decision. The risk of recurrent ICH is higher in patients with, e.g., cerebral amyloid angiopathy, hypertension, and a higher age [60]. If OAC resumption is judged necessary, DOACs could be preferred over VKA due to their lower overall risk for further hemorrhagic complications. As with recurrent IS, LAAO might be considered as an alternative treatment strategy. However, if present, we strongly prefer randomizing such patients into ongoing trials, including the Prevention of Stroke by Left Atrial Appendage Closure in Atrial Fibrillation Patients After Intracerebral Hemorrhage (STROKECLOSE) trial, to ultimately gain strong evidence on the safety and efficacy of this approach (Table 2).

## 7. Conclusions and Future Directions

AF is the most common cardiac arrythmia and by far the most important cause of cardioembolic IS. AF-related IS is usually more severe than IS due to other etiologies. As the global burden of AF is increasing rapidly due to aging populations, AF remains and will continue to be one of the most important risk factors and causal mechanisms for IS. DOACs are effective and safe in reducing the risk of IS due to AF, which makes AF screening, especially in patients with prior IS or TIA, well justified in the prevention of future IS. However, not much is thus far known about the exact mechanism underlying thrombogenesis in AF. The concepts of atrial myopathy and atrial fibrosis, and their association with AF paroxysms, should be further studied.

It is well documented that with prolonged screening more AF paroxysm will be found in patients with cryptogenic IS. As AF is often asymptomatic in IS patients, prolonged screening will likely enhance AF detection [18]. The association of these AF paroxysms found in cryptogenic IS patients is still partly unclear and should be studied in future trials. This is also true for ESUS-type IS, where prolonged monitoring techniques are used to screen patients with suspected AF. The optimal duration of screening and screening strategies with prolonged monitoring techniques should also be further explored in ESUS patients; e.g., it should be established whether a sequential screening strategy starting early with less expensive non-invasive techniques is more effective, including the total costs, compared to direct implantation of more expensive ICMs. For example, in patients with ESUS-type IS and frequent premature atrial contractions, prolonged screening with ICMs may be feasible [128]. The recent results of the ARTESiA and NOAH-AFNET 6 trials suggest that the risk of IS associated with AHRE episodes may be lower than previously thought [102,103]. In patients with subclinical AF and an increasing number of risk factors for IS and in patients with cryptogenic IS, the relationship of subclinical AF burden and risk for IS should further be studied [128]. For the time being, there is no clear consensus about the efficacy or safety of OAC in patients with short (<30 s) AF episodes [129]. 

We can expect that AI solutions and convolutional neural networks can be used to recognize patterns even from standard 12-lead ECG or in-hospital continuous ECG monitoring and with that information predict future episodes of AF and aid in recognizing patients who would benefit from more intensive rhythm monitoring to timely diagnose AF, as well as recognizing patients with the highest risk of IS with a subclinical AF burden and AHREs [130,131,132]. One aspect would be to study the role of anticoagulation in patients with a high risk of AF detected with an AI model in a randomized controlled trial. 

The optimal timing of OAC resumption has been unclear as no clear recommendations exist. The recently completed trials may prompt the update of current guidelines, but, until then, OAC resumption should be evaluated multi-disciplinarily on the basis of individual decision-making. In patients with either ischemic or hemorrhagic stroke, using OACs LAAC might be considered an alternative treatment strategy, but the overall benefit of the procedure is still unclear. Ongoing trials comparing DOACs with LAAC in patients with a recent (less than 6 months) IS are awaited to answer this question. There are also several ongoing randomized controlled trials which are studying more efficient ways to mitigate thromboembolism in patients with AF (Table 2). Finally, a newer class of OACs, factor XIa inhibitors, are under investigation and phase 3 trials are currently running to explore their efficacy and safety in IS prevention among patients with AF.

## Figures and Tables

**Table 1:** Clinical variables, laboratory imaging, and electrocardiogram biomarkers associated with a high probability of detecting atrial fibrillation. Adapted from the 2020 European Society of Cardiology Guidelines for the diagnosis and management of atrial fibrillation, and developed in collaboration with the European Association for Cardio-Thoracic Surgery; Stroke risk factors and screening [13].

Clinical Variables	
Strong prediction	Previous stroke or TIA, hypertension ageing, structural heart disease, diabetes mellitus, vascular disease, CHF/LV dysfunction, sex category (female) CHA_2_DS_2_-VASc-score (points): CHF (1), hypertension (1), age 75 years old or older (2), diabetes mellitus (1), Stroke (2), vascular disease (1), age 65–74 (1), sex category, female (1)
Weak prediction	Impaired renal function/CKD, OSA, HCM, amyloidosis in degenerative cerebral and heart diseases, hyperlipidemia, smoking, metabolic syndrome, malignancy, genetic predisposition
Laboratory biomarkers	
Blood tests	Cardiac troponin T and I, natriuretic peptides, cystatin c, CrCl/eGFR, CRP, IL-6, GDF-15, von Willebrand factor, D-dimer
Imaging and ECG biomarkers	
Echocardiogram	Left atrial enlargement or dilatation, spontaneous contrast or thrombus in LA, low LAA velocities, decreased left atrial strain, reduced left ventricular ejection fraction, complex aortic plaque
MRI or NCCT	SVD Prior or acute cortical or cerebellar infarction, multi-territory brain infarction, LVO, CMB, and white matter changes
ECG markers	Premature atrial contractions, left ventricular hypertrophy, atrioventricular block, as well as more prolonged PR interval, P-wave duration, P-wave dispersion, P-wave index, and QTc interval
ESUS-type ischemic stroke	Non-lacunar, cryptogenic ischemic strokes with proximal embolism as a probable mechanism

TIA, transient ischemic attack; CHF, chronic heart failure; LV, left ventricular; CKD, chronic kidney disease; OSA, obstructive sleep apnea; HCM, hypertrophic cardiomyopathy; CrCl, creatinine clearance; eGFR, estimated glomerular filtration rate; CRP, C-reactive protein; IL-6, interleukin 6; GDF-15, growth differentiation factor 15; LA, left atrium; LAA, left atrium appendage; MRI, magnetic resonance imaging; NCCT, non-enhanced computed tomography; SVD, small vessel disease; LVO, large vessel occlusion; CMB, cerebral microbleeds; ECG, electrocardiogram; ESUS, embolic stroke of undetermined source.

**Table 2:** Ongoing and planned trials on secondary prevention after ischemic stroke, transient ischemic attack, and intracerebral hemorrhage in patients with atrial fibrillation.

Early vs. Late Initiation of Anticoagulation after Ischemic Stroke					
Trial	Intervention	Primary Outcome	Time Frame	Study Sites	Sample Size
OPTIMAS (NCT03759938)	Early (within 4 days) vs. standard (7 to 14 days) initiation of DOAC	Stroke (ischemic or hemorrhagic) or systemic embolism.	3 months	United Kingdom	3478
Left atrial appendage closure after ischemic stroke					
Occlusion-AF (NCT03642509)	LAAC vs. DOAC within 180 after ischemic stroke	Stroke (ischemic or hemorrhagic), systemic embolism, major bleeding, or all-cause mortality.	5 years	Scandinavia	750
ELAPSE	LAAC and DOAC vs. DOAC alone	Ischemic stroke, systemic embolism, and cardiovascular death.	4 years	Not yet provided	482
LAAOS-4 (NCT05963698)	LAAC and OAC vs. OAC alone	Ischemic stroke or systemic embolism.	4 years	Not yet provided	4000
Other secondary prevention after ischemic stroke					
INTERCEPT (NCT05723926)	Bilateral carotid filter implants and OAC vs. OAC alone	Ischemic stroke.	UNK	Not yet provided	200
STABLED (NCT03777631)	Catheter ablation and OAC vs. OAC alone 1 to 6 months after ischemic stroke	Ischemic stroke, systemic embolism, all-cause death, and hospitalization for heart failure.	3 years	Japan	250
OCEANIC-AF (NCT05643573)	FXIa inhibitor (asundexian) vs. apixaban mainly as primary prevention but also includes patients with prior ischemic stroke or TIA	Stroke (ischemic or hemorrhagic) or systemic embolism.	3 years	America, Europe, Asia, Australia	18,000
LIBREXIA-AF (NCT05757869)	FXIa inhibitor (milvexian) vs. apixaban mainly as primary prevention but also includes patients with prior ischemic stroke or TIA	Stroke (ischemic or hemorrhagic) or systemic embolism.	4 years	America, Europe, Asia, Australasia, Africa	15,500
Oral anticoagulation resumption intracerebral hemorrhage					
ASPIRE (NCT03907046)	Apixaban vs. aspirin 15 to 180 days after ICH	Stroke (ischemic or hemorrhagic) or all-cause mortality. Time frame 3 years.	3 years	United States	700
ENRICH-AF (NCT03950076)	Edoxaban vs. either no antithrombotic therapy or antiplatelet monotherapy	Stroke (ischemic or hemorrhagic) or major hemorrhage.	2 years	America, Europe, Asia, Africa	1200
PRESTIGE-AF (NCT03996772)	DOAC vs. no anticoagulation 15 to 180 days after ICH	Stroke (ischemic or hemorrhagic).	3 years	Europe	350
STATICH (NCT03186729)	Anticoagulant treatment vs. no anticoagulant treatment 1 to 180 days after ICH	Fatal or non-fatal symptomatic recurrent ICH.	2 years	Scandinavia	500
Left atrial appendage closure after intracerebral hemorrhage					
A3ICH (NCT03243175)	Apixaban vs. LAAC vs. no intervention at least 14 days from ICH	Fatal or non-fatal major cardiovascular/cerebrovascular ischemic or hemorrhagic events.	2 years	France	300
STROKECLOSE (NCT02830152)	LAAO vs. medical therapy after 4 to 52 weeks after ICH	Stroke (ischemic or hemorrhagic), systemic embolism, life-threatening or major bleeding, or all-cause mortality.	5 years	Europe	750

## Data Availability

Data sharing not applicable.

## References

[1] Campbell B.C.V., Khatri P. (2020). Stroke. Lancet.

[2] Feigin V.L., Stark B.A., Johnson C.O., Roth G.A., Bisignano C., Abady G.G., Abbasifard M., Abbasi-Kangevari M., Abd-Allah F., Abedi V. (2021). Global, regional, and national burden of stroke and its risk factors, 1990–2019: A systematic analysis for the Global Burden of Disease Study 2019. Lancet Neurol..

[3] Teppo K., Airaksinen K.E.J., Jaakkola J., Halminen O., Linna M., Haukka J., Putaala J., Mustonen P., Kinnunen J., Hartikainen J. (2022). Trends in treatment and outcomes of atrial fibrillation during 2007–17 in Finland. Eur. Heart J. Qual. Care Clin. Outcomes.

[4] Choi S.E., Sagris D., Hill A., Lip G.Y.H., Abdul-Rahim A.H. (2023). Atrial fibrillation and stroke. Expert Rev. Cardiovasc. Ther..

[5] Kammersgaard L.P., Olsen T.S. (2006). Cardiovascular risk factors and 5-year mortality in the Copenhagen Stroke Study. Cerebrovasc. Dis..

[6] Lane D.A., Skjøth F., Lip G.Y.H., Larsen T.B., Kotecha D. (2017). Temporal Trends in Incidence, Prevalence, and Mortality of Atrial Fibrillation in Primary Care. J. Am. Heart Assoc..

[7] Marini C., De Santis F., Sacco S., Russo T., Olivieri L., Totaro R., Carolei A. (2005). Contribution of atrial fibrillation to incidence and outcome of ischemic stroke: Results from a population-based study. Stroke.

[8] Katsanos A.H., Kamel H., Healey J.S., Hart R.G. (2020). Stroke Prevention in Atrial Fibrillation: Looking Forward. Circulation.

[9] Lip G.Y.H., Lane D.A. (2015). Stroke Prevention in Atrial Fibrillation: A Systematic Review. JAMA.

[10] Li L., Yiin G.S., Geraghty O.C., Schulz U.G., Kuker W., Mehta Z., Rothwell P.M., Oxford Vascular Study (2015). Incidence, outcome, risk factors, and long-term prognosis of cryptogenic transient ischaemic attack and ischaemic stroke: A population-based study. Lancet Neurol..

[11] Hart R.G., Diener H.C., Coutts S.B., Easton J.D., Granger C.B., O’Donnell M.J., Sacco R.L., Connolly S.J., Cryptogenic Stroke/ESUS International Working Group (2014). Embolic strokes of undetermined source: The case for a new clinical construct. Lancet Neurol..

[12] Lehto M., Haukka J., Aro A., Halminen O., Putaala J., Linna M., Mustonen P., Kinnunen J., Kouki E., Niiranen J. (2022). Comprehensive nationwide incidence and prevalence trends of atrial fibrillation in Finland. Open Heart.

[13] Hindricks G., Potpara T., Dagres N., Arbelo E., Bax J.J., Blomström-Lundqvist C., Boriani G., Castella M., Gheorghe-Andrei D., Polychronis E.D. (2021). 2020 ESC Guidelines for the diagnosis and management of atrial fibrillation developed in collaboration with the European Association for Cardio-Thoracic Surgery (EACTS): The Task Force for the diagnosis and management of atrial fibrillation of the European Society of Cardiology (ESC) Developed with the special contribution of the European Heart Rhythm Association (EHRA) of the ESC. Eur. Heart J..

[14] Escudero-Martínez I., Morales-Caba L., Segura T. (2023). Atrial fibrillation and stroke: A review and new insights. Trends Cardiovasc. Med..

[15] Kamel H., Healey J.S. (2017). Cardioembolic Stroke. Circ. Res..

[16] Sharobeam A., Churilov L., Parsons M., Donnan G.A., Davis S.M., Yan B. (2020). Patterns of Infarction on MRI in Patients with Acute Ischemic Stroke and Cardio-Embolism: A Systematic Review and Meta-Analysis. Front. Neurol..

[17] Ntaios G. (2020). Embolic Stroke of Undetermined Source: JACC Review Topic of the Week. J. Am. Coll. Cardiol..

[18] Rubiera M., Aires A., Antonenko K., Lémeret S., Nolte C.H., Putaala J., Schabel R.B., Tuladhar A.M., Werring D.J., Zeraatkar D. (2022). European Stroke Organisation (ESO) guideline on screening for subclinical atrial fibrillation after stroke or transient ischaemic attack of undetermined origin. Eur. Stroke J..

[19] Sagris D., Harrison S.L., Buckley B.J.R., Ntaios G., Lip G.Y.H. (2022). Long-Term Cardiac Monitoring After Embolic Stroke of Undetermined Source: Search Longer, Look Harder. Am. J. Med..

[20] Kalscheur M.M., Goldberger Z.D. (2022). Screening for Atrial Fibrillation—Refining the Target. JAMA Netw. Open.

[21] Zhang J., Johnsen S.P., Guo Y., Lip G.Y.H. (2021). Epidemiology of Atrial Fibrillation: Geographic/Ecological Risk Factors, Age, Sex, Genetics. Card. Electrophysiol. Clin..

[22] Kornej J., Börschel C.S., Benjamin E.J., Schnabel R.B. (2020). Epidemiology of Atrial Fibrillation in the 21st Century: Novel Methods and New Insights. Circ. Res..

[23] Staerk L., Wang B., Preis S.R., Larson M.G., Lubitz S.A., Ellinor P.T., McManus D.D., Ko D., Weng L.C., Lunetta K.L. (2018). Lifetime risk of atrial fibrillation according to optimal, borderline, or elevated levels of risk factors: Cohort study based on longitudinal data from the Framingham Heart Study. BMJ.

[24] Krijthe B.P., Kunst A., Benjamin E.J., Lip G.Y.H., Franco O.H., Hofman A., Witteman J.C.M., Stricker B.H., Heeringa J. (2013). Projections on the number of individuals with atrial fibrillation in the European Union, from 2000 to 2060. Eur. Heart J..

[25] Lippi G., Sanchis-Gomar F., Cervellin G. (2021). Global epidemiology of atrial fibrillation: An increasing epidemic and public health challenge. Int. J. Stroke.

[26] Marcus G.M., Alonso A., Peralta C.A., Lettre G., Vittinghoff E., Lubitz S.A., Fox E.R., Levitzky Y.S., Mehra R., Kerr K.F. (2010). European ancestry as a risk factor for atrial fibrillation in African Americans. Circulation.

[27] Zoni-Berisso M., Filippi A., Landolina M., Brignoli O., D’Ambrosio G., Maglia G., Grimaldi M., Ermini G. (2013). Frequency, patient characteristics, treatment strategies, and resource usage of atrial fibrillation (from the Italian Survey of Atrial Fibrillation Management [ISAF] study). Am. J. Cardiol..

[28] Gómez-Doblas J.J., Muñiz J., Martin J.J.A., Rodríguez-Roca G., Lobos J.M., Awamleh P., Permanyer-Miralda G., Chorro F.J., Anguita M., Roig F. (2014). Prevalence of atrial fibrillation in Spain. OFRECE study results. Rev. Esp. Cardiol..

[29] Arboix A., Alió J. (2010). Cardioembolic stroke: Clinical features, specific cardiac disorders and prognosis. Curr. Cardiol. Rev..

[30] Dzeshka M.S., Lip G.Y.H., Snezhitskiy V., Shantsila E. (2015). Cardiac Fibrosis in Patients with Atrial Fibrillation: Mechanisms and Clinical Implications. J. Am. Coll. Cardiol..

[31] Kirchhof P., Breithardt G., Camm A.J., Crijns H.J., Kuck K.H., Vardas P., Wagscheider K. (2013). Improving outcomes in patients with atrial fibrillation: Rationale and design of the Early treatment of Atrial fibrillation for Stroke prevention Trial. Am. Heart J..

[32] Manolio T.A., Kronmal R.A., Burke G.L., O’Leary D.H., Price T.R. (1996). Short-term Predictors of Incident Stroke in Older Adults. Stroke.

[33] Akar J.G., Marieb M.A. (2015). Atrial Fibrillation and Thrombogenesis: Innocent Bystander or Guilty Accomplice?. JACC Clin. Electrophysiol..

[34] Watson T., Shantsila E., Lip G.Y.H. (2009). Mechanisms of thrombogenesis in atrial fibrillation: Virchow’s triad revisited. Lancet.

[35] Shen M.J., Arora R., Jalife J. (2019). Atrial Myopathy. JACC Basic Transl. Sci..

[36] Lin H.J., Wolf P.A., Kelly-Hayes M., Beiser A.S., Kase C.S., Benjamin E.J., D’Agostino B.R.B. (1996). Stroke Severity in Atrial Fibrillation. Stroke.

[37] Jørgensen H.S., Nakayama H., Reith J., Raaschou H.O., Olsen T.S. (1996). Acute stroke with atrial fibrillation. The Copenhagen Stroke Study. Stroke.

[38] Vinding N.E., Kristensen S.L., Rørth R., Butt J.H., Østergaard L., Olesen J.B., Torp-Pedersen C., Gislason G.H., Kober L., Kruuse C. (2022). Ischemic Stroke Severity and Mortality in Patients with and without Atrial Fibrillation. J. Am. Heart Assoc..

[39] Sun J., Lam C., Christie L., Blair C., Li X., Werdiger F., Yang Q., Bivard A., Lin L., Parsons M. (2023). Risk factors of hemorrhagic transformation in acute ischaemic stroke: A systematic review and meta-analysis. Front. Neurol..

[40] Olesen J.B., Lip G.Y.H., Hansen M.L., Hansen P.R., Tolstrup J.S., Lindhardsen J., Selmer C., Ahlehoff O., Schernjing Olsen A.M., Gislason G.H. (2011). Validation of risk stratification schemes for predicting stroke and thromboembolism in patients with atrial fibrillation: Nationwide cohort study. BMJ.

[41] Zhu W.G., Xiong Q.M., Hong K. (2015). Meta-analysis of CHADS2 versus CHA2DS2-VASc for predicting stroke and thromboembolism in atrial fibrillation patients independent of anticoagulation. Tex. Heart Inst. J..

[42] Joundi R.A., Cipriano L.E., Sposato L.A., Saposnik G. (2016). Ischemic Stroke Risk in Patients with Atrial Fibrillation and CHA2DS2-VASc Score of 1: Systematic Review and Meta-Analysis. Stroke.

[43] Kolominsky-Rabas P.L., Weber M., Gefeller O., Neundoerfer B., Heuschmann P.U. (2001). Epidemiology of ischemic stroke subtypes according to TOAST criteria: Incidence, recurrence, and long-term survival in ischemic stroke subtypes: A population-based study. Stroke.

[44] Granger C.B., Alexander J.H., McMurray J.J.V., Lopes R.D., Hylek E.M., Hanna M., Al-Khalidi H.R., Ansell J., Atar D., Avezum A. (2011). Apixaban versus Warfarin in Patients with Atrial Fibrillation. N. Engl. J. Med..

[45] Giugliano R.P., Ruff C.T., Braunwald E., Murphy S.A., Wiviott S.D., Halperin J.L., Waldo A.L., Ezekowitz M.D., Phil D., Weitz J.I. (2013). Edoxaban versus warfarin in patients with atrial fibrillation. N. Engl. J. Med..

[46] Hart R.G., Benavente O., McBride R., Pearce L.A. (1999). Antithrombotic therapy to prevent stroke in patients with atrial fibrillation: A meta-analysis. Ann. Intern. Med..

[47] Connolly S.J., Ezekowitz M.D., Yusuf S., Eikelboom J., Oldgren J., Parekh A., Pogue J., Reilly P.A., Themeless E., Varrone J. (2009). Dabigatran versus warfarin in patients with atrial fibrillation. N. Engl. J. Med..

[48] Patel M.R., Mahaffey K.W., Garg J., Pan G., Singer D.E., Hacke W., Breithardt G., Halperin J.L., Hankey G.J., Piccini J.P. (2011). Rivaroxaban versus Warfarin in Nonvalvular Atrial Fibrillation. N. Engl. J. Med..

[49] Ganesan A.N., Chew D.P., Hartshorne T., Selvanayagam J.B., Aylward P.E., Sanders P., MaGavigan A.D. (2016). The impact of atrial fibrillation type on the risk of thromboembolism, mortality, and bleeding: A systematic review and meta-analysis. Eur. Heart J..

[50] Friberg L., Rosenqvist M., Lip G.Y.H. (2012). Evaluation of risk stratification schemes for ischaemic stroke and bleeding in 182,678 patients with atrial fibrillation: The Swedish Atrial Fibrillation cohort study. Eur. Heart J..

[51] Curtze S., Strbian D., Meretoja A., Putaala J., Eriksson H., Haapaniemi E., Mustanoja S., Sairanen T., Satopää J., Silvennoinen H. (2014). Higher baseline international normalized ratio value correlates with higher mortality in intracerebral hemorrhage during warfarin use. Eur. J. Neurol..

[52] Borre E.D., Goode A., Raitz G., Shah B., Lowenstern A., Chatterjee R., Sharan L., Lapointe N.M.A., Yapa R., Davis J.K. (2018). Predicting Thromboembolic and Bleeding Event Risk in Patients with Non-Valvular Atrial Fibrillation: A Systematic Review. Thromb. Haemost..

[53] Dang H., Ge W.Q., Zhou C.F., Zhou C.Y. (2019). The Correlation between Atrial Fibrillation and Prognosis and Hemorrhagic Transformation. Eur. Neurol..

[54] Paciaroni M., Agnelli G., Corea F., Ageno W., Alberti A., Lanari A., Caso V., Michelli S., Bertolani L., Venti M. (2008). Early Hemorrhagic Transformation of Brain Infarction: Rate, Predictive Factors, and Influence on Clinical Outcome. Stroke.

[55] Thomas S.E., Plumber N., Venkatapathappa P., Gorantla V. (2021). A Review of Risk Factors and Predictors for Hemorrhagic Transformation in Patients with Acute Ischemic Stroke. Int. J. Vasc. Med..

[56] Hong J.M., Kim D.S., Kim M. (2021). Hemorrhagic Transformation After Ischemic Stroke: Mechanisms and Management. Front. Neurol..

[57] Paciaroni M., Bandini F., Agnelli G., Tsivgoulis G., Yaghi S., Furie K.L., Tadi P., Becattini C., Zedde M., Abdul-Rahim A.H. (2018). Hemorrhagic Transformation in Patients with Acute Ischemic Stroke and Atrial Fibrillation: Time to Initiation of Oral Anticoagulant Therapy and Outcomes. J. Am. Heart Assoc..

[58] Gokcal E., Pasi M., Fisher M., Gurol M.E. (2018). Atrial Fibrillation for the Neurologist: Preventing both Ischemic and Hemorrhagic Strokes. Curr. Neurol. Neurosci. Rep..

[59] Rosand J., Eckman M.H., Knudsen K.A., Singer D.E., Greenberg S.M. (2004). The effect of warfarin and intensity of anticoagulation on outcome of intracerebral hemorrhage. Arch. Intern. Med..

[60] Zeng Z., Chen J., Qian J., Ma F., Lv M., Zhang J. (2023). Risk Factors for Anticoagulant-Associated Intracranial Hemorrhage: A Systematic Review and Meta-analysis. Neurocrit. Care.

[61] Wilson D., Seiffge D.J., Traenka C., Basir G., Purrucker J.C., Rizos T., Sobowale O.A., Sallinen H., Yeh S.J., Wu T.Y. (2017). Outcome of intracerebral hemorrhage associated with different oral anticoagulants. Neurology.

[62] Saver J.L. (2016). Cryptogenic Stroke. N. Engl. J. Med..

[63] Tayal A.H., Tian M., Kelly K.M., Jones S.C., Wright D.G., Singh D., Jarouse J., Brillman J., Murali S., Gupta R. (2008). Atrial fibrillation detected by mobile cardiac outpatient telemetry in cryptogenic TIA or stroke. Neurology.

[64] Gladstone D.J., Spring M., Dorian P., Panzov V., Thorpe K.E., Hall J., Vaid H., O’Donnell M., Laupacis A., Lote R. (2014). Atrial Fibrillation in Patients with Cryptogenic Stroke. N. Engl. J. Med..

[65] Favilla C.G., Ingala E., Jara J., Fessler E., Cucchiara B., Messé S.R., Mullen M.T., Prasad A., Siegler J., Hutchinson M.D. (2015). Predictors of finding occult atrial fibrillation after cryptogenic stroke. Stroke.

[66] Ntaios G., Perlepe K., Lambrou D., Sirimarco G., Strambo D., Eskandari A., Karagkiozi E., Vemmou A., Koroboki E., Manios E. (2019). Prevalence and Overlap of Potential Embolic Sources in Patients with Embolic Stroke of Undetermined Source. J. Am. Heart Assoc..

[67] Camen S., Ojeda F.M., Niiranen T., Gianfagna F., Vishram-Nielsen J.K., Costanzo S., Söderberg S., Vartiainen E., Donati M.B., Lochen M.J. (2020). Temporal relations between atrial fibrillation and ischaemic stroke and their prognostic impact on mortality. EP Eur..

[68] Kirchhof P., Camm A.J., Goette A., Brandes A., Eckardt L., Elvan A., Fetsch T., van Gelder I.C., Haase D., Haegeli L.M. (2020). Early Rhythm-Control Therapy in Patients with Atrial Fibrillation. N. Engl. J. Med..

[69] Willems S., Borof K., Brandes A., Breithardt G., Camm A.J., Crijns H.J.G.M., Eckardt L., Gessler N., Goette A., Haegeli L.M. (2022). Systematic, early rhythm control strategy for atrial fibrillation in patients with or without symptoms: The EAST-AFNET 4 trial. Eur. Heart J..

[70] Perez M.V., Mahaffey K.W., Hedlin H., Rumsfeld J.S., Garcia A., Ferris T., Balasubramanian V., Russo A.M., Rajmane A., Cheung L. (2019). Large-Scale Assessment of a Smartwatch to Identify Atrial Fibrillation. New Engl. J. Med..

[71] Faranesh A., Selvaggi A.Z., Atlas C., McManus S.J., Singer D.D., Pagoto S., McConnell M.V., Pantelopoulos A., Foulkes A.S. (2022). Detection of Atrial Fibrillation in a Large Population Using Wearable Devices: The Fitbit Heart Study. Circulation.

[72] Schnabel R.B., Marinelli E.A., Arbelo E., Boriani G., Boveda S., Buckley C.M., Camm A.J., Casadei B., Chua W., Dagres N. (2023). Early diagnosis and better rhythm management to improve outcomes in patients with atrial fibrillation: The 8th AFNET/EHRA consensus conference. EP Eur..

[73] Tsivgoulis G., Palaiodimou L., Triantafyllou S., Köhrmann M., Dilaveris P., Tsioufis K., Magiorkinis G., Krogias C., Schellinger P.D., Caso V. (2023). Prolonged cardiac monitoring for stroke prevention: A systematic review and meta-analysis of randomized-controlled clinical trials. Eur. Stroke J..

[74] Svennberg E., Friberg L., Frykman V., Al-Khalili F., Engdahl J., Rosenqvist M. (2021). Clinical outcomes in systematic screening for atrial fibrillation (STROKESTOP): A multicentre, parallel group, unmasked, randomised controlled trial. Lancet.

[75] Kemp Gudmundsdottir K., Fredriksson T., Svennberg E., Al-Khalili F., Friberg L., Frykman V., Hijazi Z., Rosenqvist M., Engdahl J. (2020). Stepwise mass screening for atrial fibrillation using N-terminal B-type natriuretic peptide: The STROKESTOP II study. EP Eur..

[76] Svendsen J.H., Diederichsen S.Z., Højberg S., Krieger D.W., Graff C., Kronborg C., Olesen M.S., Nielsen J.B., Holst A.G., Brandes A. (2021). Implantable loop recorder detection of atrial fibrillation to prevent stroke (The LOOP Study): A randomised controlled trial. Lancet.

[77] Kirchhof P., Toennis T., Goette A., Camm A.J., Diener H.C., Becher N., Bertaglia E., Blomstrom Lundqvist C., Borlich M., Brandes A. (2023). Anticoagulation with Edoxaban in Patients with Atrial High-Rate Episodes. N. Engl. J. Med..

[78] Hart R.G., Sharma M., Mundl H., Kasner S.E., Bangdiwala S.I., Berkowitz S.D., Swaminathan B., Lavados P., Wang Y., Davalos A. (2018). Rivaroxaban for Stroke Prevention after Embolic Stroke of Undetermined Source. N. Engl. J. Med..

[79] Diener H.C., Sacco R.L., Easton J.D., Granger C.B., Bernstein R.A., Uchiyama S., Kreuzer J., Cronin L., Cotton D., Grauer C. (2019). Dabigatran for Prevention of Stroke after Embolic Stroke of Undetermined Source. N. Engl. J. Med..

[80] Kim A.S., Kamel H., Bernstein R.A., Manchanda M., Caprio F.Z. (2022). Controversies in Stroke: Should Patients with Embolic Stroke of Undetermined Source Undergo Intensive Heart Rhythm Monitoring with an Implantable Loop Recorder?. Stroke.

[81] Pikija S., Rösler C., Leitner U., Zellner T., Bubel N., Ganser B., Hacker C., Mutzenbach J.S. (2021). Neurologist-Led Management of Implantable Loop-Recorders After Embolic Stroke of Undetermined Source. Front. Neurol..

[82] Sposato L.A., Cipriano L.E., Saposnik G., Vargas E.R., Riccio P.M., Hachinski V. (2015). Diagnosis of atrial fibrillation after stroke and transient ischaemic attack: A systematic review and meta-analysis. Lancet Neurol..

[83] Bhat A., Mahajan V., Chen H.H.L., Gan G.C.H., Pontes-Neto O.M., Tan T.C. (2021). Embolic Stroke of Undetermined Source: Approaches in Risk Stratification for Cardioembolism. Stroke.

[84] Jiang H., Tan S.Y., Wang J.K., Li J., Tu T.M., Tan V.H., Yeo C. (2022). A meta-analysis of extended ECG monitoring in detection of atrial fibrillation in patients with cryptogenic stroke. Open Heart.

[85] Dilaveris P.E., Antoniou C.K., Caiani E.G., Casado-Arroyo R., Climent A.Μ., Cluitmans M., Cowie M.R., Doehner W., Guerra F., Jensen M.T. (2022). ESC Working Group on e-Cardiology Position Paper: Accuracy and reliability of electrocardiogram monitoring in the detection of atrial fibrillation in cryptogenic stroke patients: In collaboration with the Council on Stroke, the European Heart Rhythm Association, and the Digital Health Committee. Eur. Heart J. Digit Health.

[86] Lumikari T.J., Putaala J., Kerola A., Sibolt G., Pirinen J., Pakarinen S., Lehto M., Nieminen T. (2019). Continuous 4-week ECG monitoring with adhesive electrodes reveals AF in patients with recent embolic stroke of undetermined source. Ann. Noninvasive Electrocardiol..

[87] Lumikari T.J., Pirinen J., Putaala J., Sibolt G., Kerola A., Pakarinen S., Lehto M., Nieminen T. (2020). Prolonged ECG with a novel recorder utilizing electrode belt and mobile device in patients with recent embolic stroke of undetermined source: A pilot study. Ann. Noninvasive Electrocardiol..

[88] De Voogt W.G., van Hemel N.M., van de Bos A.A., Koïstinen J., Fast J.H. (2006). Verification of pacemaker automatic mode switching for the detection of atrial fibrillation and atrial tachycardia with Holter recording. EP Eur..

[89] Silberbauer J., Arya A., Veasey R.A., Boodhoo L., Kamalvand K., O’Nunain S., Hildick-Smith D., Paul V., Patel N.R., Lloyd G.W. (2010). The effect of bipole tip-to-ring distance in atrial electrodes upon atrial tachyarrhythmia sensing capability in modern dual-chamber pacemakers. Pacing Clin. Electrophysiol. PACE.

[90] Chen-Scarabelli C., Scarabelli T.M., Ellenbogen K.A., Halperin J.L. (2015). Device-detected atrial fibrillation: What to do with asymptomatic patients?. J. Am. Coll. Cardiol..

[91] De Voogt W.G., van Hemel N.M. (2008). Diagnostic tools for atrial tachyarrhythmias in implantable pacemakers: A review of technical options and pitfalls. Neth. Heart J..

[92] Pakarinen S., Toivonen L. (2012). Performance of atrial tachyarrhythmia-sensing algorithms in dual-chamber pacing using a fixed long AV delay in patients with sinus node dysfunction. J. Interv. Card. Electrophysiol..

[93] Kolb C., Wille B., Maurer D., Schuchert A., Weber R., Schibgilla V., Klein N., Hummer A., Schmitt C., Zrenner B. (2006). Management of far-field R wave sensing for the avoidance of inappropriate mode switch in dual chamber pacemakers: Results of the FFS-test study. J. Cardiovasc. Electrophysiol..

[94] Liu F., Yang Y., Cheng W., Ma J., Zhu W. (2021). Reappraisal of Non-vitamin K Antagonist Oral Anticoagulants in Atrial Fibrillation Patients: A Systematic Review and Meta-Analysis. Front. Cardiovasc. Med..

[95] Barold S.S., Ilercil A., Leonelli F., Herweg B. (2006). First-degree atrioventricular block. Clinical manifestations, indications for pacing, pacemaker management & consequences during cardiac resynchronization. J. Interv. Card. Electrophysiol..

[96] Glotzer T.V., Hellkamp A.S., Zimmerman J., Sweeney M.O., Yee R., Marinchak R., Cook J., Paraschos A., Love J., Radoslovich G. (2003). Atrial high rate episodes detected by pacemaker diagnostics predict death and stroke: Report of the Atrial Diagnostics Ancillary Study of the MOde Selection Trial (MOST). Circulation.

[97] Capucci A., Santini M., Padeletti L., Gulizia M., Botto G., Boriani G., Ricci R., Favale S., Zolezzi F., Di Belardinho N. (2005). Monitored atrial fibrillation duration predicts arterial embolic events in patients suffering from bradycardia and atrial fibrillation implanted with antitachycardia pacemakers. J. Am. Coll. Cardiol..

[98] Glotzer T.V., Daoud E.G., Wyse D.G., Singer D.E., Ezekowitz M.D., Hilker C., Miller C., Qi D., Ziegler P.D. (2009). The relationship between daily atrial tachyarrhythmia burden from implantable device diagnostics and stroke risk: The TRENDS study. Circ. Arrhythmia Electrophysiol..

[99] Daoud E.G., Glotzer T.V., Wyse D.G., Ezekowitz M.D., Hilker C., Koehler J., Ziegler P.D., TRENDS Investigators (2011). Temporal relationship of atrial tachyarrhythmias, cerebrovascular events, and systemic emboli based on stored device data: A subgroup analysis of TRENDS. Heart Rhythm.

[100] Brambatti M., Connolly S.J., Gold M.R., Morillo C.A., Capucci A., Muto C., Lau C.P., Van Gelder I.C., Hohnloser S.H., Carlson M. (2014). Temporal Relationship Between Subclinical Atrial Fibrillation and Embolic Events. Circulation.

[101] Turakhia M.P., Ziegler P.D., Schmitt S.K., Chang Y., Fan J., Than C.T., Keung E.K., Singer D.E. (2015). Atrial Fibrillation Burden and Short-Term Risk of Stroke: Case-Crossover Analysis of Continuously Recorded Heart Rhythm From Cardiac Electronic Implanted Devices. Circ. Arrhythmia Electrophysiol..

[102] Healey J.S., Lopes R.D., Granger C.B., Alings M., Rivard L. (2023). Apixaban for Stroke Prevention in Subclinical Atrial Fibrillation. N. Engl. J. Med..

[103] McIntyre W.F., Benz A.P., Becher N., Healey J.S., Granger C.B., Rivard L., Camm J., Goette A., Zapf A., Alings M. (2023). Direct Oral Anticoagulants for Stroke Prevention in Patients with Device-Detected Atrial Fibrillation: A Study-Level Meta-Analysis of the NOAH-AFNET 6 and ARTESiA Trials. Circulation.

[104] Hannon N., Daly L., Murphy S., Smith S., Hayden D., Ní Chróinín D., Callaly E., Horgan G., Sheehan O., Honari B. (2014). Acute hospital, community, and indirect costs of stroke associated with atrial fibrillation: Population-based study. Stroke.

[105] Hannon N., Sheehan O., Kelly L., Marnane M., Merwick A., Moore A., Kyne L., Duggan J., Moroney J., McCormack P.M.E. (2010). Stroke associated with atrial fibrillation—Incidence and early outcomes in the north Dublin population stroke study. Cerebrovasc. Dis..

[106] Kimura K., Minematsu K., Yamaguchi T. (2005). Atrial fibrillation as a predictive factor for severe stroke and early death in 15,831 patients with acute ischaemic stroke. J. Neurol. Neurosurg. Psychiatry.

[107] Fang M.C., Go A.S., Chang Y., Borowsky L.H., Pomernacki N.K., Udaltsova N., Singer D.E. (2014). Long-term survival after ischemic stroke in patients with atrial fibrillation. Neurology.

[108] Frost L., Andersen L.V., Vestergaard P., Husted S., Mortensen L.S. (2007). Trend in mortality after stroke with atrial fibrillation. Am. J. Med..

[109] Gao X., Passman R. (2022). Stroke Prevention in Atrial Fibrillation. Curr. Cardiol. Rep..

[110] Hindsholm M.F., Damgaard D., Gurol M.E., Gaist D., Simonsen C.Z. (2023). Management and Prognosis of Acute Stroke in Atrial Fibrillation. J. Clin. Med..

[111] January C.T., Wann L.S., Calkins H., Chen L.Y., Cigarroa J.E., Cleveland J.C.J., Ellinor P.T., Ezekowitz M.D., Field M.E., Furie K.L. (2019). 2019 AHA/ACC/HRS Focused Update of the 2014 AHA/ACC/HRS Guideline for the Management of Patients with Atrial Fibrillation: A Report of the American College of Cardiology/American Heart Association Task Force on Clinical Practice Guidelines and the Heart Rhythm Society. J. Am. Coll. Cardiol..

[112] Ruff C.T., Giugliano R.P., Braunwald E., Hoffman E.B., Deenadayalu N., Ezekowitz M.D., John Camm A., Weitz J.I., Lewis B.S., Parkhomenko A. (2014). Comparison of the efficacy and safety of new oral anticoagulants with warfarin in patients with atrial fibrillation: A meta-analysis of randomised trials. Lancet.

[113] Van de Werf F., Brueckmann M., Connolly S.J., Friedman J., Granger C.B., Härtter S., Harper R., Kappetein A.P., Lehr T., Mack M.J. (2012). A comparison of dabigatran etexilate with warfarin in patients with mechanical heart valves: THE Randomized, phase II study to evaluate the safety and pharmacokinetics of oral dabigatran etexilate in patients after heart valve replacement (RE-ALIGN). Am. Heart J..

[114] Piccini J.P., Caso V., Connolly S.J., Fox K.A.A., Oldgren J., Jones W.S., Gorog D.A., Durdil V., Viethen T., Neumann C. (2022). Safety of the oral factor XIa inhibitor asundexian compared with apixaban in patients with atrial fibrillation (PACIFIC-AF): A multicentre, randomised, double-blind, double-dummy, dose-finding phase 2 study. Lancet.

[115] Klijn C.J., Paciaroni M., Berge E., Korompoki E., Kõrv J., Lal A., Putaala J., Werring D.J. (2019). Antithrombotic treatment for secondary prevention of stroke and other thromboembolic events in patients with stroke or transient ischemic attack and non-valvular atrial fibrillation: A European Stroke Organisation guideline. Eur. Stroke J..

[116] Verheugt F.W.A., Ambrosio G., Atar D., Bassand J.P., Camm A.J., Costabel J.P., Fitzmaurice D.A., Illingworth L., Goldhaber S.Z., Goto S. (2019). Outcomes in Newly Diagnosed Atrial Fibrillation and History of Acute Coronary Syndromes: Insights from GARFIELD-AF. Am. J. Med..

[117] Heidbuchel H., Verhamme P., Alings M., Antz M., Hacke W., Oldgren J., Sinnaeve P., John Camm A., Kirchhof P. (2013). EHRA practical guide on the use of new oral anticoagulants in patients with non-valvular atrial fibrillation: Executive summary. Eur. Heart J..

[118] Oldgren J., Åsberg S., Hijazi Z., Wester P., Bertilsson M., Norrving B. (2022). Early Versus Delayed Non-Vitamin K Antagonist Oral Anticoagulant Therapy After Acute Ischemic Stroke in Atrial Fibrillation (TIMING): A Registry-Based Randomized Controlled Noninferiority Study. Circulation.

[119] Fischer U., Koga M., Strbian D., Branca M., Abend S., Trelle S., Paciaroni M., Thomalla G., Michel P., Nedeltchev K. (2023). Early versus Later Anticoagulation for Stroke with Atrial Fibrillation. N. Engl. J. Med..

[120] Altavilla R., Caso V., Bandini F., Agnelli G., Tsivgoulis G., Yaghi S., Furie K.L., Tadi P., Becattini C., Zedde M. (2019). Anticoagulation After Stroke in Patients with Atrial Fibrillation. Stroke.

[121] Paciaroni M., Caso V., Agnelli G., Mosconi M.G., Giustozzi M., Seiffge D.J., Engelter S.T., Lyrer P., Polymeris A.A., Kriemler L. (2022). Recurrent Ischemic Stroke and Bleeding in Patients with Atrial Fibrillation Who Suffered an Acute Stroke While on Treatment with Nonvitamin K Antagonist Oral Anticoagulants: The RENO-EXTEND Study. Stroke.

[122] Galea R., Seiffge D., Räber L. (2023). Atrial Fibrillation and Ischemic Stroke despite Oral Anticoagulation. J. Clin. Med..

[123] Seiffge D.J., De Marchis G.M., Koga M., Paciaroni M., Wilson D., Cappellari M., Macha M.D.K., Tsivgoulis G., Ambler G., Arihiro S. (2020). Ischemic Stroke despite Oral Anticoagulant Therapy in Patients with Atrial Fibrillation. Ann. Neurol..

[124] Yaghi S., Henninger N., Giles J.A., Leon Guerrero C., Mistry E., Liberman A.L., Asad D., Liu A., Nagy M., Kaushai A. (2021). Ischaemic stroke on anticoagulation therapy and early recurrence in acute cardioembolic stroke: The IAC study. J. Neurol. Neurosurg. Psychiatry.

[125] Kobeissi H., Ghozy S., Seymour T., Gupta R., Bilgin C., Kadirvel R., Rabinstein A.A., Kallmes D.F. (2023). Outcomes of Patients with Atrial Fibrillation Following Thrombectomy for Stroke: A Systematic Review and Meta-analysis. JAMA Netw. Open.

[126] Greenberg S.M., Ziai W.C., Cordonnier C., Dowlatshahi D., Francis B., Goldstein J.N., Hemphill J.D., Johnson R., Keigher K.M., Mack W.J. (2022). 2022 Guideline for the Management of Patients with Spontaneous Intracerebral Hemorrhage: A Guideline From the American Heart Association/American Stroke Association. Stroke.

[127] Dowlatshahi D., Butcher K.S., Asdaghi N., Nahirniak S., Bernbaum M.L., Giulivi A., Wasserman J.K., Poon M.C., Coutts S.B., Canadian PCC Registry (CanPro) Investigstors (2012). Poor prognosis in warfarin-associated intracranial hemorrhage despite anticoagulation reversal. Stroke.

[128] Himmelreich J.C.L., Lucassen W.A.M., Heugen M., Bossuyt P.M.M., Tan H.L., Harskamp R.E., van Etten-Jamaludin F.S., van Meert H.C.P.M. (2019). Frequent premature atrial contractions are associated with atrial fibrillation, brain ischaemia, and mortality: A systematic review and meta-analysis. EP Eur..

[129] Noseworthy P.A., Kaufman E.S., Lin Y.C., Chung M.K., Elkind M.S.V., Joglar J.A., Leal M.A., McCabe P.J., Pokorney S.D., Yao X. (2019). Subclinical and Device-Detected Atrial Fibrillation: Pondering the Knowledge Gap: A Scientific Statement From the American Heart Association. Circulation.

[130] Attia Z.I., Noseworthy P.A., Lopez-Jimenez F., Asirvatham S.J., Deshmukh A.J., Gersh B.J., Carter R.E., Yao X., Rabinstein A.A., Erickson B.J. (2019). An artificial intelligence-enabled ECG algorithm for the identification of patients with atrial fibrillation during sinus rhythm: A retrospective analysis of outcome prediction. Lancet.

[131] Noseworthy P.A., Attia Z.I., Behnken E.M., Giblon R.E., Bews K.A., Liu S., Gosse T.A., Linn Z.D., Deng Y., Yin J. (2022). Artificial intelligence-guided screening for atrial fibrillation using electrocardiogram during sinus rhythm: A prospective non-randomised interventional trial. Lancet.

[132] Khurshid S., Kartoun U., Ashburner J.M., Trinquart L., Philippakis A., Khera A.V., Ellinor P.T., Ng K., Lubitz S.A.l. (2021). Performance of Atrial Fibrillation Risk Prediction Models in Over 4 Million Individuals. Circ. Arrhythmic Electrophysiol..

